# The golgin coiled-coil proteins capture different types of transport carriers via distinct N-terminal motifs

**DOI:** 10.1186/s12915-016-0345-3

**Published:** 2017-01-26

**Authors:** Mie Wong, Alison K. Gillingham, Sean Munro

**Affiliations:** MRC Laboratory of Molecular Biology, Francis Crick Avenue, Cambridge, CB2 0QH UK

**Keywords:** Vesicle tethering, Golgin, Golgi, Coiled-coil, Endosome-to-Golgi traffic, Intra-Golgi traffic

## Abstract

**Background:**

The internal organization of cells depends on mechanisms to ensure that transport carriers, such as vesicles, fuse only with the correct destination organelle. Several types of proteins have been proposed to confer specificity to this process, and we have recently shown that a set of coiled-coil proteins on the Golgi, called golgins, are able to capture specific classes of carriers when relocated to an ectopic location.

**Results:**

Mapping of six different golgins reveals that, in each case, a short 20–50 residue region is necessary and sufficient to capture specific carriers. In all six of GMAP-210, golgin-84, TMF, golgin-97, golgin-245, and GCC88, this region is located at the extreme N-terminus of the protein. The vesicle-capturing regions of GMAP-210, golgin-84, and TMF capture intra-Golgi vesicles and share some sequence features, suggesting that they act in a related, if distinct, manner. In the case of GMAP-210, this shared feature is in addition to a previously characterized “amphipathic lipid-packing sensor” motif that can capture highly curved membranes, with the two motifs being apparently involved in capturing distinct types of vesicles. Of the three GRIP domain golgins that capture endosome-to-Golgi carriers, golgin-97 and golgin-245 share a closely related capture motif, whereas that in GCC88 is distinct, suggesting that it works by a different mechanism and raising the possibility that the three golgins capture different classes of endosome-derived carriers that share many cargos but have distinct features for recognition at the Golgi.

**Conclusions:**

For six different golgins, the capture of carriers is mediated by a short region at the N-terminus of the protein. There appear to be at least four different types of motif, consistent with specific golgins capturing specific classes of carrier and implying the existence of distinct receptors present on each of these different carrier classes.

## Background

Intracellular membrane-bound compartments are a universal feature of eukaryotic cells. Their biogenesis and function requires that lipids and proteins be able to move between the various compartments, which they do in small spherical or tubular carriers. Since different compartments have very different compositions, the maintenance of subcellular organization requires the presence of mechanisms that ensure that these transport carriers fuse only with their correct destination. The arrival of a vesicle or other carrier at a compartment is thought to occur via an initial tethering event, with subsequent closer docking of the vesicle followed by membrane fusion driven by the SNARE proteins [1–6]. Various proteins and large protein complexes have been proposed to act along this pathway and thus to contribute to specificity. However, in most cases, the relative contribution of the different proteins to specificity is unclear, as are the precise mechanisms that ensure that specificity information is recognized only when on the transport carrier and on the destination organelle.

One class of proteins that has been clearly shown to discriminate between different classes of transport carriers in vivo is the golgins, a set of long coiled-coil proteins that surround the Golgi stack [7–12]. Although transport carriers can be spherical or tubular, they are often simply referred to as vesicles, and most investigations on their generation and fusion have focused on small spherical vesicles. Thus, for simplicity, we will refer to membrane-bound carriers arriving at the Golgi as “vesicles”, but this is not intended to imply that all such carriers captured by golgins are spherical. We recently found that, when relocated to mitochondria, several different golgins are able to nucleate the tethering of distinct classes of transport vesicles that would otherwise be destined to fuse with the Golgi apparatus [13]. These golgins are recruited to different parts of the Golgi stack by their C-termini, which have either a transmembrane domain (TMD) or a domain that binds to a Golgi-localized GTPase such as Arl1 or Rab6. However, for most of the golgins, it is unclear which part of these long proteins interacts with the vesicle and how they are binding with the vesicle. One exception is GMAP-210, which has been shown to use a c40 residue stretch at its N-terminus to capture vesicles [14, 15]. This part of the protein contains an “amphipathic lipid-packing sensor” (ALPS) motif which has been shown to be able to recognize highly curved membranes in vitro, leading to the suggestion that this is how it recognizes intracellular transport vesicles [16, 17]. However, the other golgins do not have an ALPS motif and therefore other mechanisms must apply. Moreover, most of the golgins, including GMAP-210, have been found to have binding sites for Rab GTPases along their length, although it is not clear if these are required for vesicle capture [18–22].

To determine which parts of the various mammalian golgins mediate vesicle capture, we have used the mitochondrial relocation assay to map the protein components necessary for this activity and to then test if these are sufficient to confer tethering activity to a different coiled-coil protein. This approach has allowed us to show that, not only for GMAP-210, but also for golgin-84, TMF, golgin-97, golgin-245, and GCC88, a short (approximately 20–50 residue) stretch near the N-terminus is both necessary and sufficient for vesicle capture. In addition, we provide evidence to suggest that the ALPS motif is only one of two different vesicle capture activities at the N-terminus of GMAP-210, and that the three GRIP domain golgins capture vesicles by two different mechanisms.

## Results

### Golgin-84 captures intra-Golgi vesicles via its N-terminus

Golgin-84 is conserved from plants to humans and is located around the rims of the Golgi stack, where it is anchored by a C-terminal TMD [23]. When golgin-84 is relocated to mitochondria by replacing its TMD with a mitochondrial TMD, it captures vesicles that contain Golgi resident membrane proteins but not proteins coming from endosomes or the endoplasmic reticulum (ER). This indicates that golgin-84 recognizes specifically vesicles moving within the Golgi stack. To identify which part of golgin-84 is required for this recognition, we examined the ability of a series of truncated forms to capture vesicles. Although the majority of the protein is predicted to form a homodimeric coiled-coil, the first c200 residues of golgin-84 are predicted to be mostly unstructured, and we initially examined the effect of deleting this region.

**Fig. 1:**
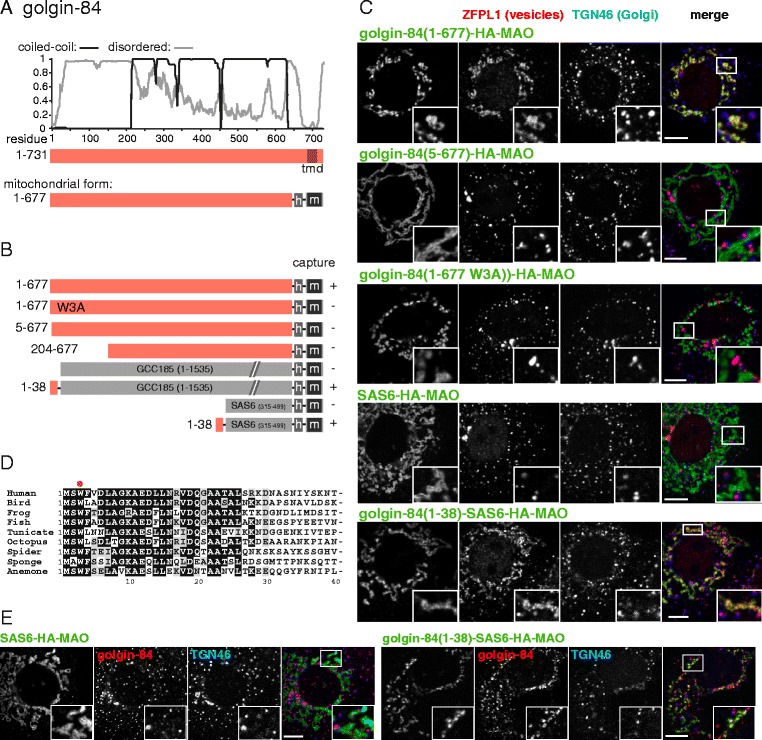
Mapping the part of golgin-84 that can capture vesicles. **a** Schematic diagram of human golgin-84 along with plots for the predicted degree of coiled-coil and disorder along its length. Also shown is the mitochondrial form in which the Golgi-targeting transmembrane domain (TMD) is replaced with a hemagglutinin (HA) tag (h) and the TMD of human monoamine oxidase A (m), a protein of the outer mitochondrial membrane. **b** Summary of the vesicle capture activity of the indicated variants of mitochondrial golgin-84. Capture at mitochondria was assayed by immunofluorescent staining of the Golgi integral membrane proteins ZFPL1, giantin and GalNAc-T2. Plus sign indicates that capture of all three markers was similar to the wild-type protein, minus sign indicates that no significant capture was observed. **c** Confocal micrographs of HeLa cells expressing the indicated golgin-84 variants and stained for the HA tag on the golgin-84 chimera as well as for ZFPL1 (in vesicles captured by golgin-84), and for TGN46 (a Golgi protein that is not captured). Cells were treated with nocodazole for 6 h prior to fixation to ensure that mitochondria were close to intra-Golgi transport vesicles. Key constructs from the set shown in (**b**) are included, with similar results obtained using the markers giantin and GalNAc-T2. Scale bars 10 μm. **d** Alignment of the N-terminus of human golgin-84 with that from the indicated species. Bird, *G. gallus*; frog, *X. laevis*; fish, *T. rubripes*; tunicate, *C. savignyi*; octopus, *O. bimaculoides*; spider, *S. mimosarum*; sponge, *A. queenslandica*; anemone, *N. vectinis*. Well conserved residues are shaded. **e** As **c** except that the cells were stained for golgin-84 using an antibody that binds outside of residues 1–38. This indicates that the N-terminus of golgin-84 is sufficient to capture vesicles containing golgin-84. Scale bars 10 μm

Vesicle capture by the golgin constructs on mitochondria was assayed by examining the intracellular distribution of the Golgi membrane proteins ZFPL1, giantin and GalNAc-T2 [13, 24–26]. Previous studies have shown that capture of intra-Golgi vesicles by golgins on mitochondria is more efficient if microtubules are depolymerized to fragment and disperse the Golgi and so ensure that it is closer to mitochondria [13, 15]. Using this approach we found that removal of the N-terminal 203 residues from golgin-84 resulted in a loss of capture of intra-Golgi vesicles (Fig. 1b). Examining smaller deletions showed that a similar loss of capture was observed with a construct lacking just the first four residues, suggesting that the most N-terminal region of the protein is particularly important (Fig. 1c). Indeed, the first c30 residues of golgin-84 are very well conserved in evolution, unlike most of the rest of the N-terminal 200 residue region (Fig. 1d). To determine if this conserved region is sufficient for vesicle capture, we initially used a mitochondrial form of GCC185, a Golgi coiled-coil protein that does not give detectable vesicle capture by fluorescence or electron microscopy in the HeLa cell line used in these assays [13, 27]. Addition of residues 1–38 of golgin-84 to the N-terminus of GCC185 was sufficient to confer vesicle capture activity to GCC185 (Fig. 1b). GCC185 is also on the Golgi, and has been implicated in vesicle transport in some systems [28, 29], and so we also attached residues 1–38 of golgin-84 to a section of coiled-coil from Sas-6, a protein from the inner part of the centriole that has no reported, or likely, link to membrane traffic [30]. When residues 315–499 of zebrafish Sas-6 were expressed with the mitochondrial localization signal they were directed to the mitochondria but showed no vesicle capture activity (Fig. 1c). However, addition of residues 1–38 of golgin-84 to Sas-6 was again sufficient to confer robust capture of Golgi membrane proteins (Fig. 1c). By using an antibody to golgin-84 that binds to a part of the protein that is C-terminal to residue 38, we were also able to show that golgin-84 is itself in the vesicles captured by golgin-84 (Fig. 1e).

Finally, we asked whether mutation of a single residue in the N-terminal region of golgin-84 is sufficient to perturb vesicle capture. Trp3 is very well conserved in golgin-84 from different species (Fig. 1d), and replacement of this single residue with alanine was sufficient to remove capture activity of the entire 677 residue cytoplasmic domain of golgin-84 (Fig. 1c). Taken together, these results show that a well conserved approximately 40-residue region at the very N-terminus of golgin-84 is both necessary and sufficient to direct the capture of intra-Golgi transport vesicles to an ectopic location.

### GMAP-210 captures intra-Golgi vesicles via its N-terminus

**Fig. 2:**
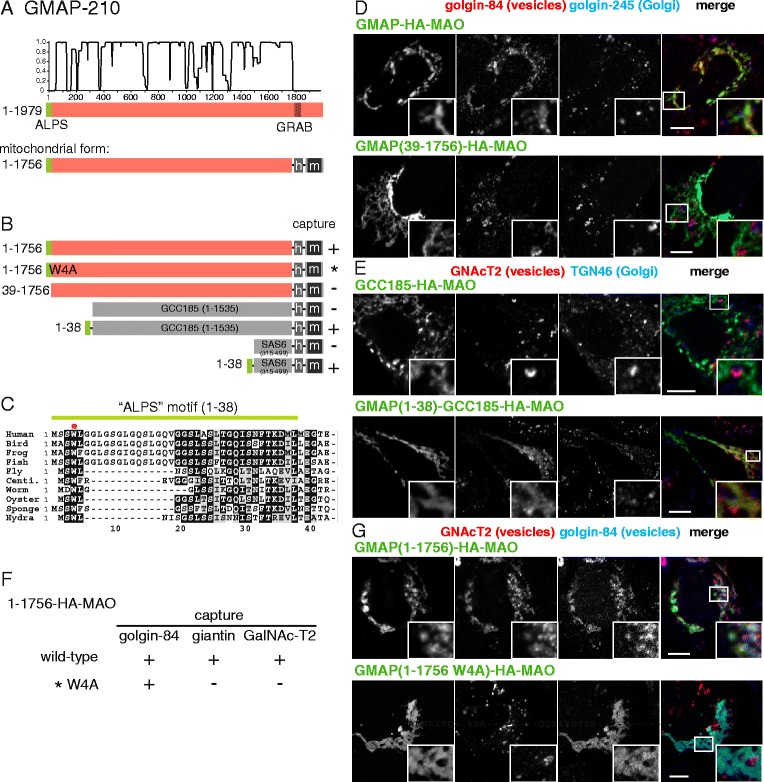
Mapping the vesicle capturing activities of GMAP-210. **a** Schematic diagram of human GMAP-210 with plots for the predicted degree of coiled-coil and disorder. In the mitochondrial form, the Golgi-targeting transmembrane domain (TMD) is replaced with a hemagglutinin (HA) tag (h) and the TMD of human monoamine oxidase A (m). **b** Summary of the vesicle capture activity of the indicated variants of mitochondrial GMAP-210. Capture at mitochondria was assayed by immunofluorescent staining of the Golgi integral membrane proteins golgin-84, giantin and GalNAc-T2. Plus sign indicates that capture of all three markers was similar to the wild-type protein, minus sign indicates that no significant capture was observed. **c** Alignment of the N-terminus of human GMAP-210 with that from the indicated species. Bird, *G. gallus*; fish *D. rerio*; frog, *X. tropicalis*; urchin, *S. purpuratus*; fly, *D. melanogaster;* centipede, *S. maritima*; worm, *S. mansoni*; oyster, *C. gigas*; sponge, *A. queenslandica*; hydra, *H. vulgaris*. Well conserved residues are shaded, and indicated as a green bar is the amphipathic lipid-packing sensor motif reported previously for the human protein [17], and by a red dot the conserved tryptophan mutated in this study. **d**, **e** Confocal micrographs of HeLa cells expressing the indicated GMAP-210 variants and stained for the HA tag on the chimera as well as for the indicated proteins in vesicles captured by GMAP-210, or for Golgi proteins that are not captured. Cells were treated with nocodazole for 6 h prior to fixation to ensure that mitochondria were close to intra-Golgi transport vesicles. Key constructs from the set shown in (**b**) are included, with similar results obtained using the marker giantin. Scale bars 10 μm. **f**, **g** As in (**d** and **e**), except comparing the complete GMAP-210 coiled-coil region with a variant in which Trp4 is mutated to alanine. This results in loss of tethering of some vesicle cargo but not golgin-84. Scale bars 10 μm

GMAP-210 is located at the cis-Golgi and has orthologs in a wide range of eukaryotes [31, 32]. It is attached to Golgi membranes via a C-terminal domain that binds Arf1 [16, 33]. When this domain in human GMAP-210 is replaced with a mitochondrial targeting signal, the mitochondrial GMAP-210 captures vesicles containing Golgi resident membrane proteins, and also shows transient capture of ER to Golgi carriers [13]. GMAP-210 is predicted to form a dimeric coiled-coil over most of its length (Fig. 2a). Near the N terminus, there is an ALPS motif that has been shown in vitro to preferentially bind small liposomes rather than large ones, suggesting that it can detect the high curvature of vesicles [17]. Use of the mitochondrial relocation assay has shown that the first 38 residues, including the ALPS motif, are required for vesicle tethering by GMAP-210 [15]. Whether this region is sufficient for vesicle capture has not been reported, although the N-terminal 38 residues of GMAP-210 have been shown to be sufficient to capture small liposomes when displayed on the mitochondria of permeabilized cells, and also to be sufficient to localize to the Golgi region when expressed in cells as a fusion to a heterologous coiled-coil [14]. We thus tested the effect of expressing on mitochondria chimeric proteins containing parts of GMAP-210. As expected, removal of the N-terminal 38 residues resulted in a loss of the ability to capture vesicles containing Golgi resident proteins such as GalNAc-T2, ZFPL1, golgin-84 and giantin (Fig. 2c, d). When residues 1–38 were attached to a heterologous coiled-coil protein they were sufficient to confer vesicle capture activity (Fig. 2b, e).

Alignment of the N-terminal regions of GMAP-210 from diverse species illustrates the ALPS motif as conserved regularly-spaced hydrophobic residues that would form a hydrophobic face on one side of an α-helix (Fig. 2c). However, it is also striking that there are some residues in this region that appear invariant between species rather than simply preserving a hydrophobic character. In addition, part of the putative ALPS motif is absent in non-vertebrate species even though the conserved residues that flank this region are still present. When the best conserved residue, Trp4, was mutated to alanine, capture of GalNAc-T2 and giantin was lost but capture of golgin-84 could still be observed (Fig. 2f, g). This striking observation suggests that, in addition to the ALPS motif, the N-terminal region of GMAP-210 makes a sequence-specific interaction with a sub-population of vesicles containing GalNAc-T2 and giantin, whereas this is less relevant to a different population of vesicles containing golgin-84 but not GalNAc-T2 and giantin. Taken together, these results suggest that the ALPS motif and the conserved residues that flank it are able to capture vesicles by two different mechanisms and that these two features make a differential contribution to each mechanism, with a vesicle class that contains golgin-84 but not GalNAc-T2 depending less on the conserved N-terminal motif containing the tryptophan.

### TMF captures intra-Golgi vesicles via both the N-terminus and also a central portion of its coiled-coil

TMF is well conserved across eukaryotic phyla and has been reported to be localized on the rims of the Golgi towards the trans-side [34–36]. Null mutations in mice show defects in the formation of the Golgi-associated acrosome in sperm, and perturbations in the heavily glycosylated mucin layer of the intestine [37, 38]. TMF is recruited to the Golgi via a C-terminal Rab6-binding domain [35]. When relocated to mitochondria, TMF captures intra-Golgi transport vesicles, but these contain some proteins from later in the stack than those captured by GMAP-210 and golgin-84. For instance, all three capture GalNAc-T2, but only TMF captures ST6GalT while only GMAP-210 and golgin-84 capture ZFPL1.

**Fig. 3:**
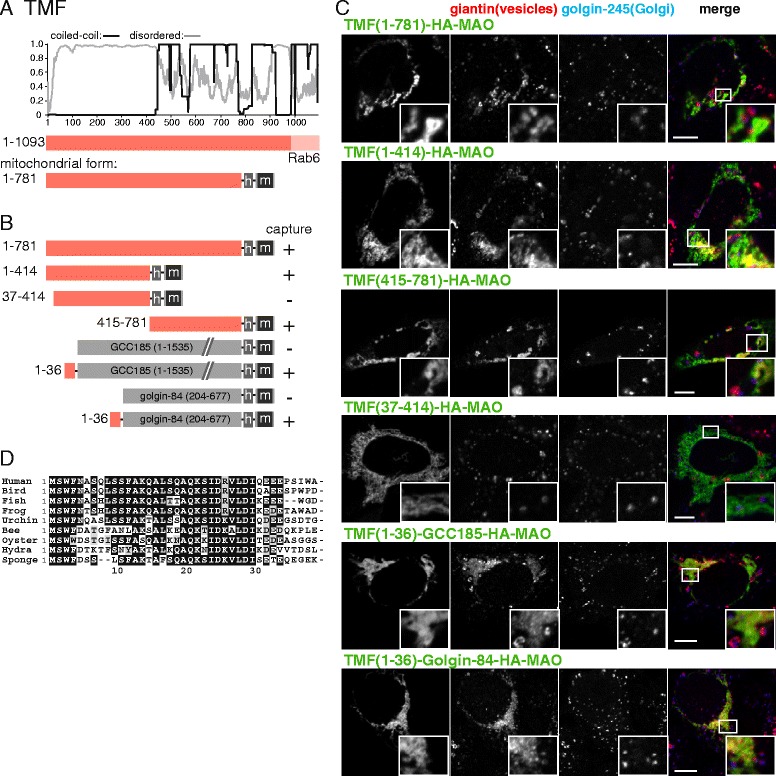
Mapping the vesicle capturing activity of TMF. **a** Schematic diagram of human TMF with plots for the predicted degree of coiled-coil and disorder. In the mitochondrial form, the Golgi-targeting transmembrane domain (TMD) is replaced with a hemagglutinin (HA) tag (h) and the TMD of human monoamine oxidase A (m). **b** Summary of the vesicle capture activity of the indicated variants of mitochondrial TMF. Capture at mitochondria was assayed by immunofluorescent staining of the Golgi integral membrane proteins golgin-84, giantin, and GalNAc-T2. Plus sign indicates that capture of all three markers was similar to the wild-type protein, minus sign indicates that no significant capture was observed. **c** Confocal micrographs of HeLa cells expressing the indicated TMF variants and stained for the HA tag on the golgin-84 chimera as well as for giantin that is in vesicles captured by TMF and for golgin-245, a protein that remains Golgi associated. Cells were treated with nocodazole for 6 h prior to fixation to ensure that mitochondria were close to intra-Golgi transport vesicles. Key constructs from the set shown in (**b**) are included, with similar results obtained using the markers golgin-84 and GalNAc-T2. Scale bars 10 μm. **d** Alignment of the N-terminus of human TMF with that from the indicated species. Bird, *G. gallus*; frog, *X. tropicalis*; fish *D. rerio*; urchin, *S. purpuratus*; bee, *A. mellifera*; oyster, *C. gigas*; hydra, *H. vulgaris*; sponge, *A. queenslandica*

TMF is predicted to be unstructured for most of an approximately 400-residue region at the N-terminus, with this followed by approximately 500 residues of coiled-coil (Fig. 3a). To map the vesicle capture activity, we expressed truncations of TMF as mitochondrial forms. Surprisingly, both the unstructured N-terminal region and also the C-terminal coiled-coil were sufficient for vesicle capture (Fig. 3b, c). The vesicle capture activity within the coiled-coil region has proven difficult to map, perhaps due to truncations destabilizing the coiled-coil homodimer, but vesicle capture activity within this region is at least consistent with previous reports that this part of the protein can bind to the Golgi when the COPI vesicle coat is removed [39]. We were, however, able to map the capture activity in the N-terminal half, which again has a particularly well conserved N-terminal region (Fig. 3d). The first 36 residues of the protein are necessary for the capture activity of the N-terminal half, and sufficient to confer capture activity when attached to two different heterologous coiled-coil proteins (Fig. 3b, c). Thus, like the other golgins, TMF has vesicle capture activity in a conserved N-terminal region, but appears distinct in also having readily detectable capture activity elsewhere in the protein.

### GRIP domain golgin GCC88 captures vesicles via an N-terminal motif

GCC88 is one of four human golgins that share a C-terminal GRIP domain that binds to the trans-Golgi GTPase Arl1, the others being GCC185, golgin-97, and golgin-245 [40–42]. Most metazoans have orthologs of all four proteins, with non-metazoans typically having only one GRIP domain golgin [43, 44]. The proteins have been strongly implicated in traffic from endosomes to Golgi, and we were able to detect the capture of endosome-to-Golgi carriers by mitochondrial forms of three of them, the exception being GCC185, with capture by GCC88 being somewhat less efficient than that by the other two [13, 45, 46].

**Fig. 4:**
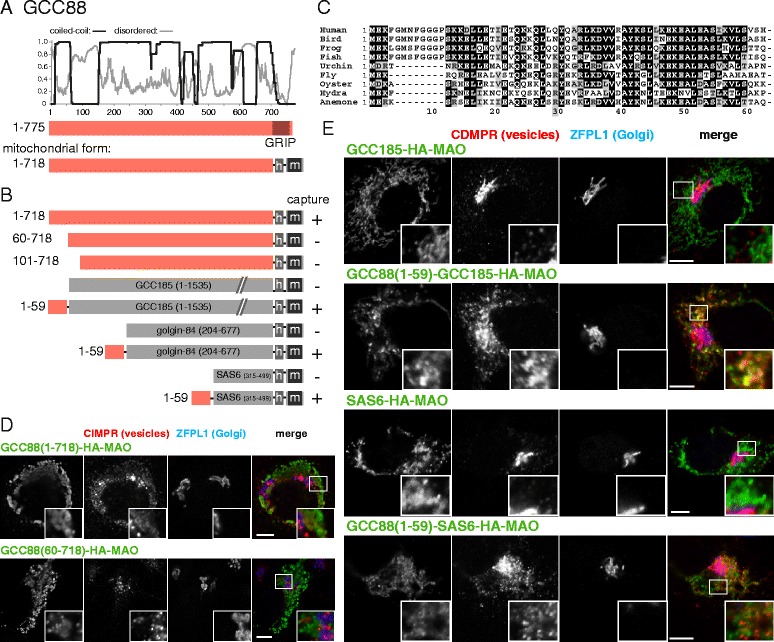
Mapping the part of GCC88 that can capture vesicles. **a** Schematic diagram of human GCC88 along with plots for the predicted degree of coiled-coil and disorder along its length. Also shown is the mitochondrial form as in Fig. 1a. **b** Summary of the vesicle capture activity of the indicated truncations and chimeras of mitochondrial GCC88. Capture at mitochondria was assayed by immunofluorescent staining of the integral membrane proteins CD-MPR, CI-MPR and Vti1a. A plus sign indicates that capture of all three markers was similar to the wild-type protein, a minus sign indicates that no significant capture was observed. **c** Alignment of the N-terminus of human GCC88 with that from the indicated species. Bird, *G. gallus*; frog, *X. tropicalis*; fish *D. rerio*; urchin, *S. purpuratus*; fly, *D. melanogaster;* oyster, *C. gigas*; hydra, *H. vulgaris*; anemone, *N. vectinis*. **d**, **e** Confocal micrographs of HeLa cells expressing the indicated GCC88 variants and stained for the hemagglutinin tag on the GCC88 chimera as well as for both CD-MPR (in vesicles captured by GCC88) and ZFPL1 (a cis-Golgi protein that is not captured). Key constructs from the set shown in (**b**) are included, with similar results also obtained using the vesicle markers CI-MPR and Vti1a. Scale bars 10 μm

To map the vesicle-capturing region of GCC88, we expressed various forms of the protein, all with the GRIP domain replaced with a mitochondrial TMD. Most of GCC88 is predicted to be coiled-coil with a c100 residue stretch near the N-terminus predicted to be disordered (Fig. 4a). Removal of the first 100 residues resulted in a complete loss of the ability of GCC88 to relocate to mitochondria endosome-to-Golgi cargo such as the cation-dependent and the cation-independent mannose 6-phosphate receptors (CD-MPR and CI-MPR, respectively), and the SNARE Vti1a (Fig. 4b). The N-terminal 60 residues are particularly well conserved between species, and when this region was deleted, vesicle capture activity was lost (Fig. 4c, d). Moreover, when residues 1–59 were attached to mitochondrial forms of three different heterologous coiled-coil proteins, in every case, the resulting chimeras were able to capture endosome-to-Golgi cargo (Fig. 4b, e). Taken together, these data show that a conserved, approximately 60-residue, region at the N-terminus of GCC88 is necessary and sufficient to capture carriers containing endosome-to-Golgi cargo.

### The GRIP domain golgins golgin-97 and golgin-245 both capture vesicles via an N-terminal motif

**Fig. 5:**
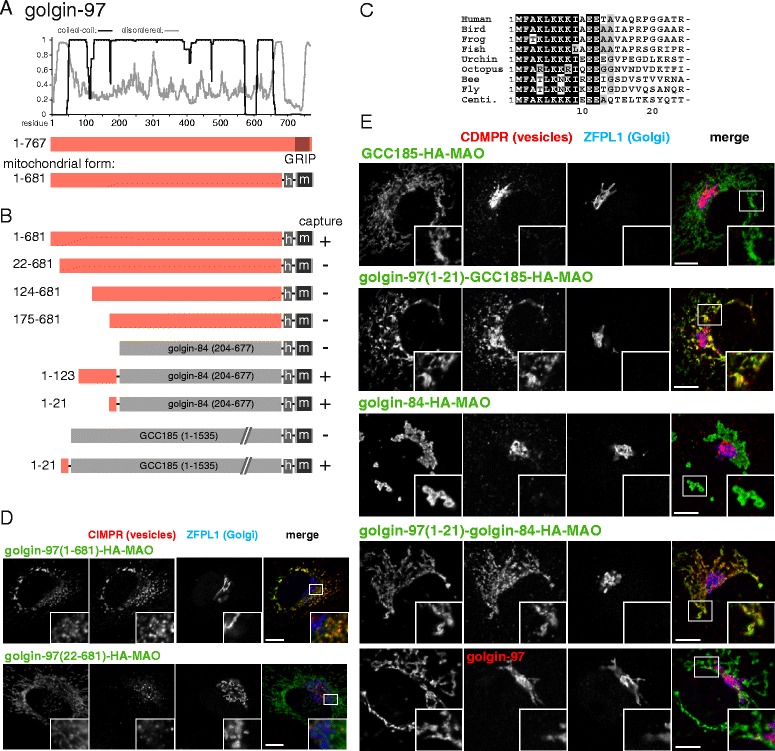
Mapping the part of golgin-97 that captures vesicles. **a** Schematic diagram of human golgin-97 along with plots for the predicted degree of coiled-coil and disorder along its length. Also shown is the mitochondrial form in which the GRIP domain has been replaced with a hemagglutinin (HA) tag and the human monoamine oxidase A transmembrane domain. **b** Summary of the vesicle capture activity of the indicated truncations and chimeras of golgin-97. Capture at mitochondria was assayed by immunofluorescent staining of the integral membrane proteins CD-MPR, CI-MPR and Vti1a. A plus sign indicates that capture of all three markers was similar to the wild-type protein, a minus sign indicates that no significant capture was observed. **c** Alignment of the N-terminus of human golgin-97 with that from the indicated species. Bird, *G. gallus*; frog, *X. tropicalis*; fish *D. rerio*; urchin, *S. purpuratus*; octopus, *O. bimaculoides*; bee, *A. mellifera*; fly, *D. melanogaster;* centipede, *S. maritime*. **d**, **e** Confocal micrographs of HeLa cells expressing the indicated golgin-97 variants and stained for the HA tag on the golgin-97 chimera as well as for CI-MPR or CD-MPR (in vesicles captured by golgin-97) along with ZFPL1 (a cis-Golgi protein that is not captured). Key constructs from the set shown in (**b**) are included, with similar results obtained using the vesicle markers CD-MPR, CI-MPR or Vti1a. Scale bars 10 μm

In addition to GCC88, two further GRIP domain golgins are able to capture endosome-to-Golgi cargo. These are golgin-97 and golgin-245, and, like GCC88, they are well conserved amongst metazoans [13, 45, 46]. We initially examined truncations of golgin-97 attached to mitochondria and found that removal of the N-terminal 123 or 174 residues resulted in loss of capture activity (Fig. 5a, b). Of these 123 residues, the first c20 are particularly well conserved and indeed the first 21 residues were required for activity (Fig. 5c, d). Consistent with this, the first 21 residues were sufficient to confer capture activity to two different heterologous coiled-coil proteins (Fig. 5e). Interestingly, an antibody to golgin-97 that recognizes residues C-terminal to residue 21 revealed that endogenous golgin-97 is not relocalized by these chimeric proteins (Fig. 5e). This confirms that the membranes being captured by mitochondrial golgin-97 are endosome-derived transport vesicles rather than fragments of TGN as has been suggested elsewhere [11, 47]. Taken together, these results indicate that a short well-conserved region at the N-terminus of golgin-97 is necessary and sufficient to nucleate the capture of endosome-to-Golgi carriers.

**Fig. 6:**
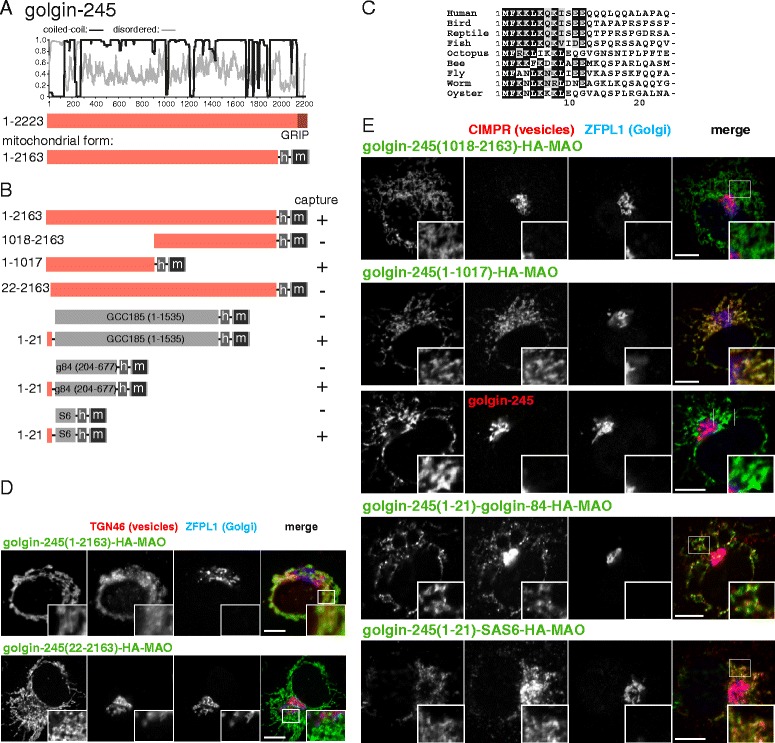
Mapping the part of golgin-245 that captures vesicles. **a** Schematic diagram of human golgin-245 along with plots for the predicted degree of coiled-coil and disorder along its length. Also shown is the mitochondrial form as in Fig. 1a. **b** Summary of the vesicle capture activity of the indicated truncations and chimeras of golgin-245. Capture at mitochondria was assayed by immunofluorescent staining of the integral membrane proteins CD-MPR, CI-MPR, TGN46 or Vti1a. A plus sign indicates that capture of all four markers was similar to the wild-type protein, a minus sign indicates that no significant capture was observed. **c** Alignment of the N-terminus of human golgin-245 with that from the indicated species. Bird, *G. gallus*; reptile, *A. mississippiensis*; fish *D. rerio*; octopus, *O. bimaculoides*; bee, *A. mellifera*; fly, *D. melanogaster*; worm, *C. elegans*; oyster, *C. gigas*. **d**, **e** Confocal micrographs of HeLa cells expressing the indicated golgin-245 variants and stained for the hemagglutinin tag on the golgin-245 chimera as well as for either TGN46 or CI-MPR (in vesicles captured by golgin-245) as well as for ZFPL1 (a cis-Golgi protein that is not captured). Key constructs from the set shown in (**b**) are included, with similar results obtained using the vesicle markers CI-MPR, TGN46, CD-MPR, and Vti1a. Scale bars 10 μm

The final GRIP domain golgin that can capture endosome-to-Golgi cargo when relocated to mitochondria is golgin-245 [13, 48]. Although longer than the others, it is still predicted to be coiled-coil over most of its length with an approximately 100 residue unstructured region at the N-terminus (Fig. 6a). Removal of the first 1017 residues resulted in loss of capture activity, whilst removal of all residues past 1017 did not have this effect (Fig. 6b, e). Like the other golgins, the N-terminal region is particularly well conserved (Fig. 6c), and indeed the first 21 residues were found to be necessary for capture activity (Fig. 6b, d). As with golgin-97 this 21 residue region was sufficient to confer capture activity on three different heterologous coiled-coil proteins (Fig. 6b, e). It was also possible to use an antibody to golgin-245 that binds C-terminal to residue 21 to show that golgin-245 itself was not relocated by the golgin-245 N-terminus, adding further support to the argument that capture of CD-MPR and other endosome-to-Golgi cargo is not simply a reflection of capture of fragments of the TGN (Fig. 6e). Thus, the vesicle capture of this long protein is encoded in a short N-terminal region that represents just one percent of its total length.

### The vesicle capturing sequences of the different golgins fall into families

**Fig. 7:**
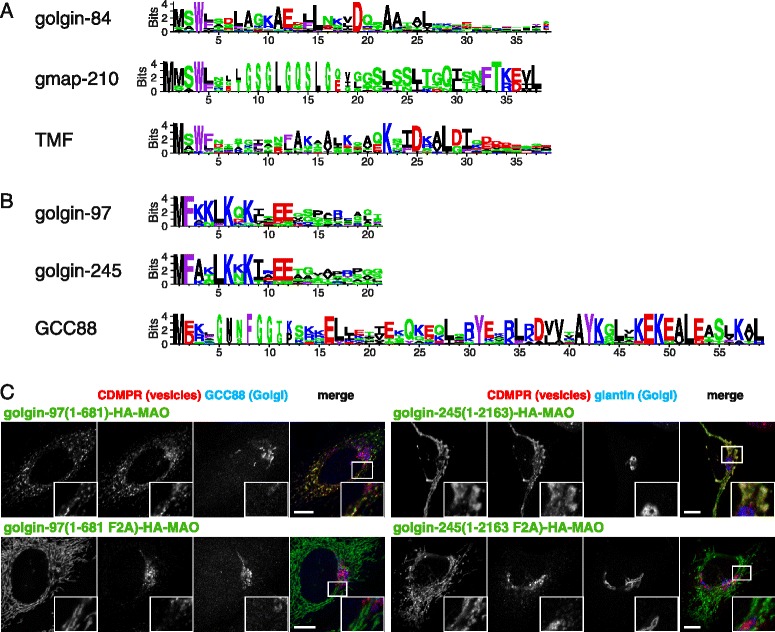
Patterns of conserved residues in the golgin vesicle capturing motifs. **a** Logo plots of the N-terminal regions of the three golgins that capture intra-Golgi transport vesicles. The height of the residue indicates how well it is conserved in orthologs from diverse metazoans, expressed as information content (bits). Residues are colored by their properties as follows: *red*, basic; *blue*, acidic; *green*, polar but uncharged; *black*, aliphatic; and *purple*, aromatic. **b** Logo plots of the N-terminal regions of the three GRIP domain golgins that capture endosome - to - Golgi transport vesicles. Residues as in (**a**). **c** Confocal micrographs of HeLa cells expressing the full - length mitochondrial forms of golgin-97 or golgin-245 or variants in which the conserved Phe2 residue is mutated to alanine, and stained for the hemagglutinin tag on the chimera. In both cases, this mutation results in loss of tethering of vesicles as indicated by CD-MPR. Scale bars 10 μm

We have been able to show that, for six of the mammalian golgins, the key region for vesicle capture resides at the N-terminus. To compare the regions from the different proteins, we generated “logo” plots that highlight residues that are well conserved between different species (Fig. 7). These plots confirm that the N-terminal region of each golgin contains residues that are particularly well conserved between species. In addition, comparing the patterns of conserved residues from the different golgins reveals two striking patterns.

The first pattern revealed by the logo plots is that the three golgins that capture intra-Golgi vesicles (golgin-84, GMAP-210 and TMF) share a short motif (M-S-W-L/F) at the very N-terminus, even though they differ elsewhere with, for instance, GMAP-210 having the ALPS motif (Fig. 7a). The M-S-W-L/F motif includes a tryptophan that we have shown to be important for tethering activity for both golgin-84 and GMAP-210. This suggests that even if the three proteins do not recognize identical features on vesicles, they may well recognize related features.

The second pattern emerging from comparing these logo plots is that the vesicle capture motifs of two of the GRIP domain golgins, golgin-97 and golgin-245, are very similar to each other but clearly distinct from that of GCC88 (Fig. 7b). This shared motif contains a particularly well conserved phenylalanine in the second position and for both golgin-97 and golgin-245 mutating this residue to alanine resulted in a loss of tethering activity. Taken together, these observations suggest that golgin-97 and golgin-245 capture vesicles by a very similar, if not identical, mechanism. In contrast, it seems quite possible that GCC88 uses a different mechanism for vesicle capture, and thus there may be at least two classes of vesicles delivering cargo from endosomes to Golgi, with one of these classes being captured by golgin-97 and golgin-245 and the other by GCC88.

## Discussion and conclusions

The finding that seven of the known golgins are able to redirect specific Golgi-bound carriers to an ectopic location raises many questions about the mechanisms underlying vesicle capture at the Golgi. Our work, combined with a previous analysis of GMAP-210, has revealed that, for six of the seven golgins, the vesicle capture occurs via a short conserved region at the very N-terminus of the protein. The two golgins that are exceptions are TMF and GM130. For TMF, it seems that a region in the middle of the coiled-coil section also has efficient capture activity, but TMF is an unusual golgin in that its coiled-coil section is much better conserved than that of the others, suggesting that this part of the protein may have a unique structure or additional functions. GM130 is one of two golgins that capture ER to Golgi carriers, the other being GMAP-210 [13]. This activity is transient and technically challenging to assay over a range of constructs and so a detailed analysis of the capture of these carriers will form the basis of a future study. However, it is known that a conserved region at the N-terminus of GM130 binds directly to p115, a protein implicated in the tethering of vesicles in both yeast and mammals [49–51]. This suggests that at least part of GM130’s activity resides in a conserved N-terminal region even if there may also be capture activity elsewhere. As such, it is clear that most, if not all, of the golgins that capture vesicles do so via a short N-terminal region even though the type of vesicle captured varies between the golgins. Capture at the N-terminus being a common feature of golgin function raises several interesting questions of mechanism.

**Fig. 8:**
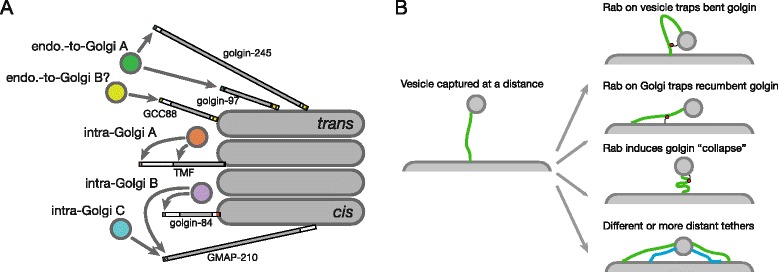
Summary of vesicle capture by golgins. **a** Summary of the vesicle capture activities of the six indicated golgins. Golgin-97 and golgin-245 seem likely to capture the same type of vesicle, whilst the region of GCC88 that has capture activity has a very different sequence and therefore either captures the same vesicle by a different mechanism, or a different type of vesicle with overlapping cargo. The remaining three golgins capture intra-Golgi vesicles, and it appears that those bound to TMF have a similar but not identical set of cargo to those captured by golgin-84. There appear to be two different types of vesicle captured by GMAP-210. The precise number of classes of intra-Golgi vesicle, however, remains unclear. In all cases, the vesicle is captured by an N-terminal motif, with TMF also having a capture activity in its coiled-coil region. **b** Some of the possible models for how a vesicle captured at the N-terminus of a golgin could move closer to the Golgi membrane so as to allow vesicle fusion. The Rab binding sites on the golgin could be used in various ways to hold the golgin N-terminus closer to the membrane, or to induce a conformational change in the golgin. Alternatively, the vesicle could be captured by a further, more distant, golgin, or by different, shorter golgins (*blue*), to hold the vesicle closer to the target membrane. Of course, all these models are speculative, and other models could also apply

Firstly, it is clear that, although a similar part of the protein is involved in each case, the mechanism of action is likely to be different as the conserved sequence, and the type of vesicle recognized, are not the same for the different proteins. Indeed, our analysis has revealed further complexity than was apparent from earlier studies as GMAP-210 is apparently able to capture two different classes of intra-Golgi transport vesicle, and golgin-97 and golgin-245 capture a class of endosome-to-Golgi carrier that may be distinct from that caught by GCC88 (summarized in Fig. 8a). This indicates that the golgins will be useful tools for discriminating between different populations of transport vesicles so as to investigate their content and the machinery required for their generation.

A second question is what is being bound by these N-terminal sequences. Although it is known that most of the golgins have binding sites for Rab GTPases, these sites are found along the length of the coiled-coil and in no case thus far reported do they correspond to the vesicle binding regions at the N-termini [18, 19, 21]. It seems likely that different vesicles are marked by at least five different types of golgin binding-features. Four seem unambiguous, the first being that recognized by GCC88, the second by the golgin-97/245 pair, the third by the ALPS motif, and the fourth by the MSWF/L-containing parts of GMAP-210, golgin-84, and TMF. Moreover, the differences in the N-termini of golgin-84 and TMF outside of the MSWF region suggest that these proteins could recognize two distinct, if related, features. The next challenge will be to identify the molecules that bind directly to these vesicle capture regions, and the mapping reported here should expedite this process.

The third question raised by this work is why the golgins apparently only bind vesicles efficiently at the point furthest from where they are attached to the target membrane – an arrangement that might be expected to slow rather than assist fusion. This finding suggests that the movement of the captured vesicle toward the target membrane is not simply mediated by there being similar binding sites for the same vesicle feature arrayed along the length of the golgin. It is formally possible that there are weaker vesicle binding sites along the length of the golgin that cannot be readily detected in our relocation assay, and indeed at expression levels higher than those used in the experiments described herein, we have observed weak capture by some of the constructs even in the absence of their N-terminal motifs. However, there are also other mechanisms that could move the captured vesicles closer to the target membrane and hence the SNAREs and other factors that direct membrane fusion (Fig. 8b). Coiled-coil proteins are in general thought to be somewhat flexible, although the persistence length of the golgins themselves has not been determined. In addition, coiled-coil predictions for the golgins suggest that there are short breaks in their coiled-coil regions, and it has long been suggested that these could be flexible hinges that help the golgins to bend [10, 52, 53]. Such a bent conformation could be stabilized by Rabs on the vesicle or the target membrane binding to the golgin itself (Fig. 8b). Alternative possibilities are that a Rab interaction initiates a structural alteration in the coiled-coil so that it collapses to bring vesicle and organelles together [47, 54], or that interactions with more distant golgins, or shorter golgins, pull the vesicle closer to the membrane (Fig. 8b).

Clearly, there are many further questions that need to be addressed before we have a full understanding of the mechanism by which golgins contribute to membrane traffic. The multitude of vesicle capture mechanisms used by the golgins that we reveal in this study suggests that investigating these questions will reveal a rich diversity of new information about the underlying logic of cellular organization.

## Methods

### Protein sequence analysis

Coiled-coil prediction plots were generated with COILS using a 28 residue window [55], and disorder prediction plots generated using DISOPRED3 [56]. Relatives of the human golgins from different species were identified with BLAST, aligned with Clustal Omega [57], and shaded with BoxShade. Logo plots were generated using the Shannon method in Seq2Logo 2.0 [58].

### Plasmids

N-terminal deletions and truncations of the golgins were constructed by PCR-amplification using C-terminally truncated golgins as templates or by In-Fusion cloning and all were then inserted upstream of a hemagglutinin tag and the C-terminal TMD of human monoamine oxidase A (residues 481–527) in pcDNA3.1+ (Clontech) [13]. In all cases, an initiator methionine was included at the start of the coding sequence. C-terminally truncated template golgins were as follows: GCC88ΔC-term (1-Ala^718^); GCC185ΔC-term (1-Ser^1535^); golgin-97ΔC-term (1-Val^681^); golgin-245ΔC-term (1-Gly^2163^); TMFΔC-term (1-Thr^781^); golgin-84ΔC-term (1-Ala^677^); and GMAP-210ΔC-term (1-Leu^1756^). Site-directed mutagenesis was performed using overlapping primers containing the required mutation followed by DpnI digestion, and verified by DNA sequencing.

### Antibodies

Antibodies used in this study were mouse CD-MPR (1/100, 22d4, Developmental Studies Hybridoma Bank (DSHB)), mouse GalNAc-T2 (neat, UH-4, gift from U. Mandel and H. Clausen), rabbit GCC88 (1/300, HPA021323, Sigma), mouse CI-MPR (1/100, ab2733, Abcam), rabbit giantin (1/300, HPA011008, Sigma), mouse GM130 (1/300, 610823, BD Biosciences), mouse golgin-245 (1/200, 611281, BD Biosciences), rabbit golgin-84 (1/300, HPA000992, Sigma), rat HA (1/300, 3 F10, Roche), sheep TGN46 (1/300, AHP500, AbD serotec), rabbit TMF (1/300 HPA008729, Sigma), and rabbit ZFPL1 (1/500, HPA014909, Sigma).

### Cell culture, transient transfections, and treatments

HeLa cells (ATCC) were cultured in Dulbecco’s modified Eagle’s medium (Invitrogen) supplemented with 10% fetal calf serum (FCS) and penicillin/streptomycin at 37 °C and 5% CO_2_. Cells were tested for mycoplasma contamination (MycoAlert, Lonza). Cells were transiently transfected with Fugene6 (Promega) according to the manufacturers’ instructions. Cells plated on six-well plates were transfected 12–24 h after plating when they had reached 50–80% confluency, and then analyzed 24–48 h following transfection. To depolymerize microtubules, cells were treated for 6 h at 37 °C with 0.5 μM nocodazole (Sigma, from a 3.3 mM stock in DMSO), prior to fixation.

### Immunofluorescence

HeLa cells split onto multi-well glass slides 24 h prior to fixation were fixed with 4% formaldehyde in PBS for 20 min, permeabilized in 0.5% Triton X-100 for 10 min, and blocked in blocking buffer (20% FCS, 0.5% Tween-20 in PBS) for 30–60 min. Primary and secondary antibodies (Alexa Fluor, Invitrogen) were applied in blocking buffer for 1 h; cells were washed five times with PBS and mounted under a cover slip in Vectashield mounting medium (Vector Labs). To assess the various truncated forms, we examined at least three markers that were affected by the full-length mitochondrial form, and at least three that were not, examining at least 20 transfected cells for each combination. All construct/marker combinations that are illustrated were assessed in independent experiments by two observers. Images were acquired with a Zeiss LSM780 confocal microscope using a Plan-Apochromat 63X oil-immersion objective.
